# Behavioral Factors of Longevity

**DOI:** 10.4061/2011/197590

**Published:** 2012-01-22

**Authors:** Peter Martin, Leonard W. Poon, Bo Hagberg

**Affiliations:** ^1^Department of Human Development and Family Studies, Iowa State University, Ames, IA 50011, USA; ^2^Insitute of Gerontology, University of Georgia, Athens, GA 30602, USA; ^3^Department of Psychology, Lund University, 22220 Lund, Sweden

The majority of studies on longevity have focused disproportionally on biomedical aspects of longevity [1]. While biomedical aspects undoubtedly play an important role in determining the length and quality of life, there are also a number of important social, psychological, and behavioral factors associated with longevity. Recent research has demonstrated that distal experiences such as education [2] and childhood personality [3] as well as proximal behaviors such as nutritional behaviors [4], coping with stress [5], and social support [6] are all important components in determining mortality, longevity, and quality of life among very old people.

This special issue on behavioral factors of longevity attempts to highlight important behavioral factors associated with longevity. We pose two important questions. The first question concerns predictors of longevity. Why do some people live to a very long life whereas others do not? The second question concerns the quality of life for individuals who have lived a very long life. What is life like when one reaches nonagenarian or centenarian status? We were, therefore, guided by two separate but interrelated aspects of longevity research: studies discussing factors contributing to longevity and studies discussing behavioral aspects among long-lived individuals.

The articles in the special issue cover a number of important topics. The first highlights the importance of biopsychosocial models in longevity research (Picard). With regard to the mortality and survivorship aspect, papers include gender differences (M. Poulain et al.; F. Balard et al.), minority representation and socioeconomic status (L. S. Ka'opua et al.; Yao and Robert), season of birth (Gavrilov and Gavrilova), obesity (Cohen-Mansfield and Perach; J. Nocera et al.), alcohol consumption (E. K. Howie et al.), dental health (A. Paganini-Hill et al.), personality (B. P. Chapman et al.), stress trajectories (C. M. Aldwin et al.), depression and cognition (D. Paulson et al.), and cognitive beliefs (Fry and Debats). The second topic of life quality includes the importance of dietary patterns (D. B. Hausman et al.), physical and psychological well-being (J. Cho et al.), social support (Ailshire and Crimmins; G. K. Randall et al.), and life satisfaction (A. J. Bishop et al.).

It is clear from these contributions that there are many behavioral factors contributing to longevity. Broadly speaking, they include health and health behaviors, individual characteristics, such as gender, ethnicity, and socioeconomic status, stress, cognitive beliefs, and cognition, as well as social and environmental support, mental health, and life satisfaction. In a previous publication, we included all these factors in the Georgia adaptation model [7]. An extension of this model would include important distal variables.

The studies included in this volume highlight and introduce specific behaviors that are associated with longevity. Surely, there might be other specific behaviors of importance for longevity that are not covered here but the components proposed to be of importance in this issue allow us to take a much broader perspective on aging that goes beyond biomedical explanations. We hope this special issue will stimulate more research on behavioral factors of longevity.

*Peter Martin* *Peter Martin* *Leonard W. Poon* *Leonard W. Poon* *Bo Hagberg* *Bo Hagberg*
